# Structural biology of SMC complexes across the tree of life

**DOI:** 10.1016/j.sbi.2023.102598

**Published:** 2023-06

**Authors:** Frank Bürmann, Jan Löwe

**Affiliations:** MRC Laboratory of Molecular Biology, Structural Studies Division, Cambridge Biomedical Campus, Cambridge, CB2 0QH, UK

**Keywords:** SMC complexes, DNA loop extrusion, DNA entrapment, Chromosome organization

## Abstract

Structural maintenance of chromosomes (SMC) complexes guard and organize the three-dimensional structure of chromosomal DNA across the tree of life. Many SMC functions can be explained by an inherent motor activity that extrudes large DNA loops while the complexes move along their substrate. Here, we review recent structural insights into the architecture and conservation of these molecular machines, their interaction with DNA, and the conformational changes that are linked to their ATP hydrolysis cycle.

## Highlights

• Side-by-side comparison of SMC holo-complex architectures.
• Overview of DNA binding compartments.
• Structural insights into SMC complex dimerization.

## Introduction

**Figure 1:**
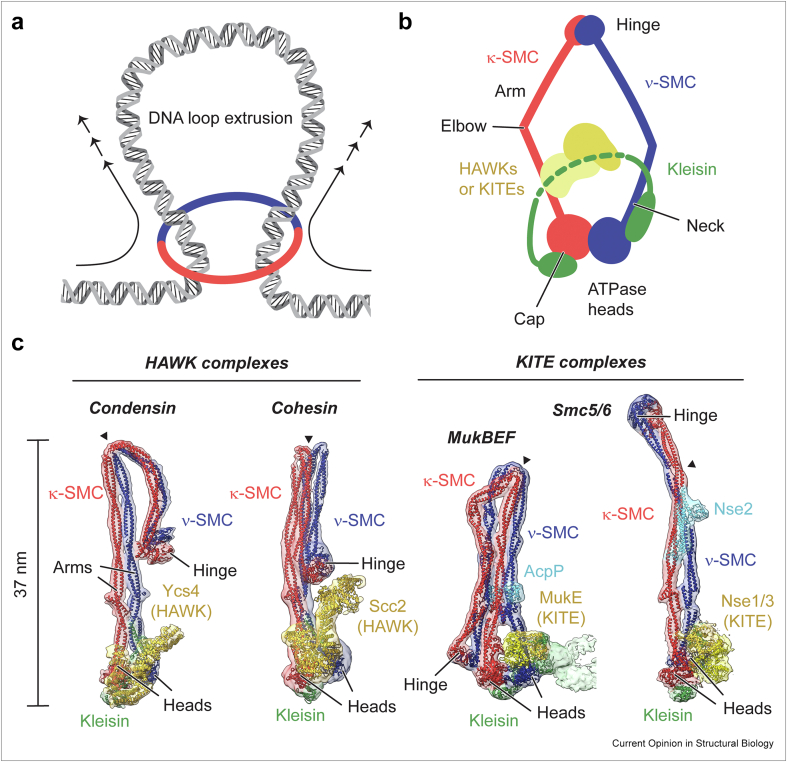
(**a**) DNA loop extrusion by an SMC complex. (**b**) Subunit composition and basic architecture of SMC complexes. The schematic is shown in the familiar “open ring” representation. (**c**) Cryo-EM structures of HAWK- and KITE-based SMC complexes in their ATP/DNA-free apo form. The position of an elbow/coiled-coil discontinuity is marked with a black arrowhead.

Large-scale three-dimensional rearrangements of chromosomal DNA drive and facilitate diverse genomic processes, from chromosome segregation to gene expression, DNA repair, and recombination. SMC complexes are deeply involved in these conformational transitions in eukaryotes and prokaryotes. Multiple lines of evidence, from chromosome conformation studies [1,2] to single-molecule experiments [3, 4, 5, 6∗], suggest that SMC complexes achieve this at least in part by an intrinsic motor that extrudes large loops of DNA (Figure 1a). The motor is driven by an ATPase residing in the SMC head domains (Figure 1b), related to ATP binding cassette (ABC) transporters. SMC heads dimerize upon ATP binding, which enables hydrolysis of the nucleotide. The heads then dissociate and release the hydrolysis products, resetting the enzyme to its “apo” state. This cycle of head “engagement” and “disengagement”, fuelled by the consumption of ATP, propels the conformational changes that implement DNA loop extrusion.

The SMC head is attached to a long intramolecular coiled-coil “arm”, which ends in a dimerization domain called “hinge” (Figure 1b). The hinge holds two SMC proteins together, which are also bridged by a “kleisin” subunit close to their heads [7]. The kleisin connects the coiled-coil “neck” of one SMC subunit (ν-SMC; Greek “nu” for neck) to the ATPase “cap” of the second SMC subunit (κ-SMC; Greek “kappa” for cap). It also provides binding sites for peripheral subunits, which either belong to the HAWK (HEAT protein associated with a kleisin) or KITE (kleisin interacting tandem winged-helix element) protein families [8,9]. HAWK-based SMC complexes have so far been identified exclusively in eukaryotes (condensin and cohesin). In contrast, KITE-based complexes are found in both eukaryotes (Smc5/6) and prokaryotes (Smc–ScpAB, MukBEF/MksBEF/Wadjet).

In this short review, we summarize recent structural findings relating to the architecture of SMC complexes and their interaction with DNA, highlighting publications from the past two years (2021 and 2022). We refer the reader to excellent complementary resources for further reading and a detailed overview of current functional models [10,11].

## The architecture of SMC holo-complexes

Electron cryomicroscopy (cryo-EM) methods now permit the structural analysis of intact SMC holo-complexes in different functional states, and in several cases visualized the spatial relationships of all subunits and domains simultaneously. Structures of condensin in its apo and ATP-bound states [12] and cohesin in a DNA-bound state [13, 14, 15] have been complemented by structures of condensin in a DNA-bound form [16,17], and cohesin in an apo state [18]. In addition to these HAWK-based complexes, structures of the KITE-based complexes MukBEF [19], Smc5/6 [20,21], and MksBEF/Wadjet [22,23] were obtained in apo and DNA-bound states. These findings now give us structural snapshots of SMC complexes across the phylogenetic tree, separated by a billion-year timespan of evolution.

The findings have revealed multiple shared and distinct architectural principles (Figure 1c). Generally, in the apo state, the coiled-coil arms closely align from hinge to head in a “zipped-up” conformation. The ATPase heads are disengaged but close, separated only by a shallow cleft. This restricts access to the DNA binding site on top of the heads, which is split by head disengagement. In condensin, cohesin, and MukBEF, the arms fold back at an “elbow”, which brings the hinge domain closer to the heads and peripheral subunits. Although overall similar in size and shape, these folded conformations show a large degree of variation. In condensin, the hinge is kept high up on the ν-SMC side of the complex, whereas in cohesin it is positioned more centrally and closer to the heads. In MukBEF, the hinge is brought all the way down near the heads and is positioned at the κ-SMC side, opposite to what is observed for condensin. All is in contrast to Smc5/6, which adopts an extended rod-like shape that keeps its hinge far away from the heads. The Smc5/6 arm contains a central coiled-coil discontinuity that contributes to its slight curvature [20,24]. This region is close to the binding site of the SUMO ligase subunit Nse2. While Nse2 is essential for viability, its ligase activity is not [25], suggesting an additional structural or regulatory role perhaps in stabilizing the extended rod. Partial reconstruction of a bacterial MksBEF/Wadjet complex suggests that its elbow adopts an angle close to 90°, halfway between folded and extended configurations [22].

In cohesin, back-folding allows the hinge to contact the DNA-binding STAG1/Scc3 subunit [13,26] and may enable similar interactions with Scc2–4/NIPBL–MAU2 and its substitute Pds5 [14,18,27]. In the other SMC complexes, an association of the hinge with the head-proximal subunits seems geometrically difficult. This architectural diversity suggests that SMC complexes may leverage the back-folded conformation for specialized purposes.

## DNA binding and entrapment

**Figure 2:**
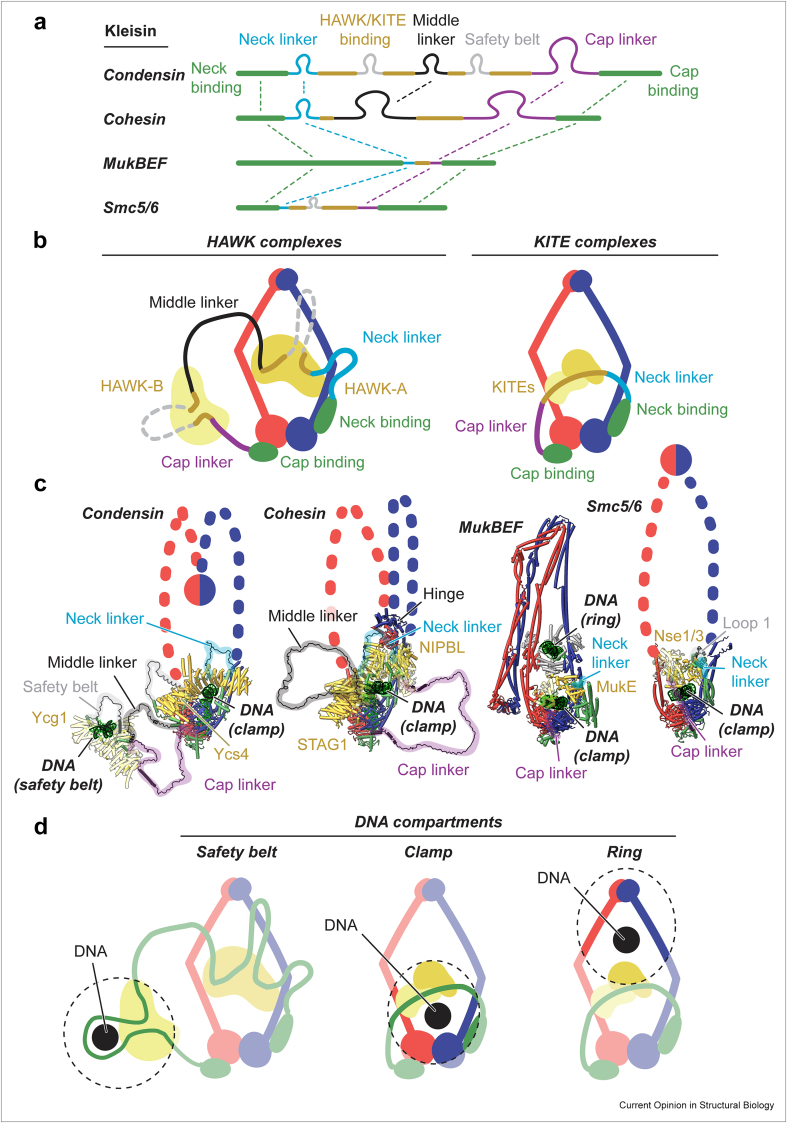
(**a**) Comparison of kleisin chains. (**b**) The schematic architecture of HAWK and KITE complexes, with kleisin regions coloured as in A. (**c**) Comparison of DNA clamped structures. Flexible loop regions not resolved are indicated as black segments. Unresolved arms and hinges are shown as schematics. (**d**) DNA compartments observed by structural methods.

One hallmark activity of SMC complexes is the “entrapment” of DNA, whereby the complex fully encircles its substrate. The kleisin subdivides the complex into different compartments that can entrap DNA (Figure 2). Some of these compartments are remodelled during the ATPase cycle, which then controls their loading with DNA.

The kleisins of the HAWK complexes contain several long and flexible linkers that are either much shorter or absent in the KITE-associated kleisins (Figure 2a). Binding sites for the peripheral subunits are in the central region of the kleisin and connect to the N- and C-terminal SMC binding domains via the neck and cap linker, respectively (Figure 2b). The HAWK binding sites in condensin and cohesin are connected by a long and flexible “middle linker”, which is absent from the KITE-associated kleisins of MukBEF and Smc5/6. Condensin's kleisin, together with its Ycg1 subunit, entraps DNA in a “safety belt” adjacent to the middle linker [16,17,28]. This is thought to provide a stable anchor during DNA loop extrusion [3,17]. Although cohesin's middle linker could allow the formation of a similar compartment, this has not been reported so far. The kleisins of MukBEF and Smc5/6 lack the middle linker and are thus unable to form a safety belt compartment. Their cap and neck linkers are also short and constrained, and likely cannot wrap around DNA.

Upon ATP-mediated dimerization, the SMC heads bind DNA on their top surface (Figure 2c). The kleisin passes over the head-bound DNA, guiding HAWKs or KITEs to hold the DNA in the “clamp” compartment. HAWKs and KITEs are unrelated but serve analogous roles, and a similar type of DNA binding has been observed in the SMC-like Rad50–Mre11/SbcCD [29]. Among HAWK complexes, the clamp stoichiometry seems to differ. Two HAWKs are part of the cohesin clamp, namely NIPBL/Scc2 and STAG1/Scc3. While the clamp does not strictly require STAG1/Scc3 for DNA binding [15], it is additionally held in place by contacts of STAG1/Scc3 with the hinge [13]. In contrast, only a single HAWK is part of the condensin clamp, namely Ycs4 [16,17]. The other HAWK, Ycg1, is flexibly tethered and constrains the safety belt. In the KITE-based MukBEF complex, the dimeric KITE MukE binds DNA parallel to its symmetry axis [19], whereas in Smc5/6, DNA clamping is mostly mediated by the Nse3 KITE and much less by its partner Nse1 [21]. The structure of a MksBEF/Wadjet complex bound to DNA revealed an “unclamped” conformation, whereby DNA is also bound on top of the heads, but both kleisin and KITEs are positioned below the heads and do not contribute to DNA binding [22]. Remodelling of the clamp by the ATPase cycle becomes apparent in the apo structures: the clamp compartment is empty, and closed by aligned arms, disengaged heads, and displaced HAWKs/KITEs (Figure 1c). In conclusion, the clamp has considerable structural plasticity, is remodelled by the ATPase, and is likely (with the possible exception of MksBEF/Wadjet) a core compartment of SMC complexes.

Another compartment common among SMC complexes is the large “ring”, which is delineated by the SMC arms, the hinge, and the kleisin/HAWK/KITE subcomplex (Figure 2**d**). In the structure of MukBEF, a second DNA is also entrapped in this compartment [19]. This DNA is bound by MatP, which is thought to flag DNA for unloading [30,31]. Topological cross-linking experiments of MukBEF are consistent with continuous loop formation between ring- and clamp-bound DNA, and topological mapping of Smc5/6 supports a similar type of loop formation [32].

A less-well understood mode of DNA binding is mediated by some SMC hinge domains, which have been reported to bind both double- and single-stranded DNA. Binding of single-stranded DNA may be involved in the DNA repair functions of Smc5/6 and replication-coupled loading of cohesin [33,34].

## DNA gating and DNA topology

The entrapment and release of DNA require the opening of DNA gates. Recent topological cross-linking studies of cohesin using reconstituted DNA loading reactions suggest that DNA can enter via two gates [35]: DNA can pass through dissociated hinge domains, or an open neck gate, whereby the kleisin N-terminal domain detaches from the Smc3 neck. Loading through the hinge appears to be the major pathway. Similar to cohesin [26,36], condensin's neck gate opens upon ATP binding when DNA is absent [16]. Regulation of this interface by microcephalin/MCPH1 was suggested to mediate DNA unloading [37], similar to the opening of cohesin's neck gate by WAPL [38]. Smc5/6 opens its neck gate upon binding of the loader Nse5–6 [32], the neck gate of MukBEF forms a cleft in the presence of ATP, DNA, and the unloader MatP [19], and the MksBEF/Wadjet complex drastically repositions its neck gate upon the formation of the unclamped state [22,23]. The remodelling of the neck gate has thus emerged as a feature common among SMC complexes.

Reconstituted loop extrusion reactions on DNA with large, immobilized obstacles suggested that both condensin and cohesin can bypass sub-micrometre-sized objects [39]. In the case of cohesin, bypassing was still observed after covalent cross-linking of the hinge and kleisin gates, indicating that it did not depend on gate opening. This may suggest that DNA in these reactions is not entrapped at all. Alternatively, DNA may shuttle exclusively into compartments not closed by the cross-links (for example the clamp) or bypassing of obstacles may create more complex DNA structures, such as secondary loops, that are not resolved by light microscopy [17].

Recent *in vitro* studies revealed that DNA loop extrusion by condensin is enhanced by positive supercoiling [40] and that the complex can restrain short negatively supercoiled loops under similar conditions [41]. Moreover, MksBEF/Wadjet complexes involved in the bacterial defence against plasmids were shown to recognize circular DNA to activate their associated nuclease MksG/JetD [22,23]. These findings reinforce the notion that SMC function is intimately linked to DNA conformation and topology.

## Conformational changes during the ATPase cycle

Several recent studies have investigated the conformational transitions that take place during the ATP hydrolysis cycle. An opening of the SMC arms upon ATP binding was observed in the bacterial Smc–ScpAB complex using electron paramagnetic resonance experiments [42]. Cross-linking of the arms interfered with ATPase activation upon DNA binding or locked the complex in a continuously activated state. Genetic studies of yeast cohesin have identified point mutations in the arms that rescue ATPase-deficient mutants [43]. Both studies suggested that arm conformations and the ATPase cycle are functionally linked. Some ATPase regulators also bind the coiled-coil arms. The acyl carrier protein AcpP associates with the head-proximal arms of MukBEF to activate its ATP turnover [19,44]. Nse5–6 was shown to inhibit the ATPase of Smc5/6 [45,46], and parts of its binding site were mapped to the head-proximal arm [24,46]. Interestingly, recent structures of Nse5–6 show that Nse6 is a HEAT repeat protein similar to the HAWKs, although it lacks their characteristic hook shape [24,46]. If not coincidental, this may point towards another evolutionary link between the HAWK- and KITE-based SMC complexes.

Magnetic tweezer experiments with loop-extruding condensin revealed that the complex compacts DNA in steps of several hundred base pairs [47]. The experiments suggested that ATP binding rather than hydrolysis may be sufficient for a single compaction step. This indicates that condensin undergoes a considerable conformational change upon binding of DNA and nucleotide. Consistent with this notion, the arms in the available DNA-clamped structures of condensin were not resolved [16,17], suggesting at least increased flexibility compared to the apo state.

Recent FRET studies on yeast cohesin indicate that Scc3/STAG1 stays associated with the hinge throughout the ATPase cycle, whereas Scc2/NIBPL does not [48]. Hinge-bound Scc3/STAG1 may thus serve as a DNA shuttle during a “Brownian ratchet” swing-out of the arms. Single-molecule FRET and AFM studies on human cohesin have observed a swing-out upon ATP binding, moving the hinge away from the heads [49]. In contrast to the observations made for yeast, NIPBL/Scc2 instead of STAG1/Scc3 appeared to stay associated with the hinge, possibly transporting DNA by a “swing-and-clamp” motion. Conceivably, the reported swing-out is at odds with the ATP-bound cryo-EM structures of cohesin, where the hinge is observed near the heads. Thus, it is possible that the structures only represent a subset of several possible states.

We note that DNA transport by a hinge-bound shuttle is likely a cohesin-specific phenomenon and seems unfeasible for KITE-based complexes. It would require long kleisin linkers for long-distance movements, which are absent from these complexes. The “hold-and-feed” model proposed for condensin [17] does not involve a DNA shuttle but also relies on long kleisin linkers, which are absent from the KITE complexes. Several variations of the related “segment-capture” model [50] do not necessitate long linkers but require an external “anchor” element or SMC complex dimerization to explain DNA loop extrusion.

## SMC complex dimerization

Single-molecule loop-extrusion experiments of condensin suggest that the complex acts as a monomer [3]. In contrast, human cohesin was reported to act as either a monomer [5] or a dimer [4]. A recent study reconstituted DNA loop extrusion by a KITE complex, Smc5/6, which was observed in a dimeric state [6].

**Figure 3:**
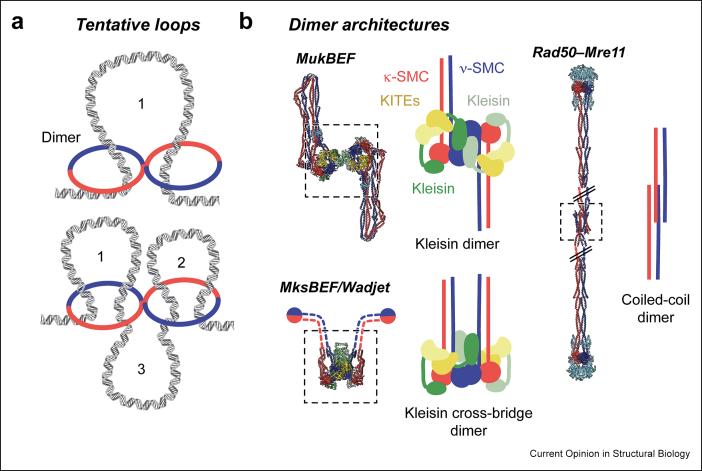
(**a**) Tentative loop formation by dimeric SMC complexes. (**b**) Dimers of SMC complexes as observed by cryo-EM. Dimerization can be mediated by the kleisin (MukBEF and MksBEF/Wadjet), or by the coiled-coil arms (Rad50–Mre11). Note that the different arrangements position the KITEs on the front of the dimer (MukBEF), or both front and back (MksBEF/Wadjet). Rad50–Mre11 is distantly related to SMC complexes and does not contain KITEs/HAWKs/kleisins. Dashed lines and black double lines indicate missing density and shortening of the displayed region, respectively.

Dimeric SMC complexes may enable bidirectional loop extrusion, perhaps proceeding via a single loop or multiple coupled loops (Figure 3a). The cryo-EM structures of MukBEF and MksBEF/Wadjet show examples of how SMC complexes can dimerize [19,22,23]. MukBEF dimerizes via the N-terminal domains of its kleisin, with the KITE subunits on one side of the dimer (Figure 3b). MksBEF/Wadjet, in contrast, forms a kleisin cross-bridge, whereby the kleisin connects SMC proteins of separate complexes, positioning the KITEs on opposite sides of the dimer. An entirely different mode of dimerization was observed in a recent cryo-EM structure of the SMC-like Rad50–Mre11, which is held together at its coiled coils [51]. These findings highlight that at least some SMC and SMC-like complexes form dimers mediated by protein–protein contacts, but also suggest that there may be considerable plasticity in how this is achieved. An overview of shared and distinct SMC features is shown in Table 1.

**Table 1:** Features of SMC complexes. This picture could change since not all states have been investigated to exhaustion, and new states remain to be discovered.

Feature	Condensin	Cohesin	MukBEF	Smc5/6	Wadjet
HAWK	+	+			

KITE			+	+	+

Arm back-folding	+	+	+		

Safety belt	+				

Long kleisin linkers	+	+			

Clamp/ring compartments	+	+	+	+	

Neck gate remodelling	+	+	+	+	+

Kleisin dimerization			+		+

## Conclusions

Rapid progress in the cryo-EM imaging of SMC complexes has given us new insights into their architecture, conservation, and interactions with DNA. These snapshots of a sophisticated reaction cycle will be useful for designing and interpreting future experiments and models. We anticipate ongoing efforts to further clarify the relationship between DNA entrapment and loop extrusion, how DNA conformation and topology are used or sensed, and how exactly the ATPase cycle powers DNA transport. Overall, it is fascinating to see the conservation and diversity of these proteins that have served our genomes for billions of years.

## Conflict of interest statement

Nothing declared.

## Data Availability

No data was used for the research described in the article.
